# The Recent Grave Robbery in This City

**Published:** 1874-10

**Authors:** 


### Editorial.

### The Recent Grave Robbery in this City.

The excitement over the desecration of the grave at Lime Stone Hill, on the night of October 5th, has not yet subsided in some portions of the city. Many people are still unwilling to believe that the body taken from the College on Friday morning was not that of Mrs. Carey, although her physicians, last in attendance, positively assert that it was not. It would hardly seem probable to a thoughtful person, however, that the College authorities would run the risk which the raising of a body from Lime Stone Hill or any other cemetery would entail, and it seems still more improbable that after the warning contained in the papers they would allow the body, if in their possession, to remain in the open dissecting room. We were considerably surprised at the action of Mr. Carey in removing the body under dispute, for it was proved conclusively that it was not the body of his late wife. Mrs. Carey died, we are assured by three honest and well known physicians, of cancer of the uterus, of such an extensive character that the recto-vaginal septum was wholly destroyed, and fæces passed through the vagina as well as through the anus. In the case of the body under dispute, no evidence of cancer could be found, but the post mortem plainly evinced that the cause of death was Gastroenteritis, of which there was not the slightest symptom in Mrs. Carey's case; moreover the body found in the dissecting room had undergone such *post mortem* changes that it was perfectly evident that death had taken place at a much earlier period than October 4th.

We did not take up our pen, however, to defend the College or the profession, but after a careful consideration of the facts, we hope that no sensible person will allow himself to make the absurd statement that "the profession will stand by each other, and of course they would swear that this was not the body stolen from Mrs. Carey's grave." We are glad the public have so high an opinion of the profession, but we are surprised that after making such a statement regarding p ofessional honor they should immediately doubt professional honesty, and even decla e that they would swear to a lie.

